# Effect of Coenzyme Q_10_ Supplementation on Testosterone

**DOI:** 10.3390/biom8040172

**Published:** 2018-12-13

**Authors:** Saleem Ali Banihani

**Affiliations:** Department of Medical Laboratory Sciences, Jordan University of Science and Technology, Irbid 22110, Jordan; sabanihani@just.edu.jo; Tel.: +962-2-7201000

**Keywords:** coenzyme Q_10_, testosterone, antioxidant, oxidative stress, luteinizing hormone, reproductive toxicity

## Abstract

Enhancing testosterone production in males is a continuous research direction for many scientists in the field, due to its role as a principal sex hormone and as a crucial modulator of well-being and general health in humans. Since 1978, there have been more than 30 studies that have connected coenzyme Q_10_ and testosterone. Such a link is attributable to the vigorous biological role of coenzyme Q_10_ as a crucial member in the energy production route in humans and animals, which is thought to have a positive influence on testosterone production, and hence on infertility, particularly male infertility. However, this connection has not yet been deliberated. The present work systematically reviews and summarizes the influence of coenzyme Q_10_ supplementation on testosterone. To accomplish this purpose, the Scopus, PubMed, and Web of Science databases were searched using the keywords “coenzyme Q_10_” versus “testosterone” for English language papers from November 1978 through October 2018. Relevant articles were also discussed and included to address an integral discussion. In summary, to date the studies conducted on human males reveal insignificant effects of coenzyme Q_10_ supplementation on testosterone. Similarly, rather than the reproductive toxicity studies, the studies conducted on animals did not show any positive influence of coenzyme Q_10_ on testosterone. However, coenzyme Q_10_ supplementation was found to ameliorate the reduction in testosterone induced by chemical reproductive toxicants, mainly by neutralizing the damaging effect of the generated free radicals. However, collectively these findings require further confirmation by additional research studies.

## 1. Introduction

**Figure 1:**
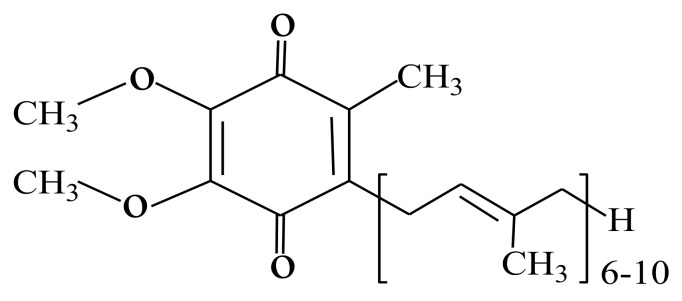
Chemical structure of coenzyme Q_10_.

Coenzyme Q_10_ (1,4-benzoquinone) or ubiquinone, most of the time abbreviated as CoQ10, is a ubiquitous lipid-soluble biomolecule present in the mitochondria of almost all animals and bacteria [1]. The letter Q refers to the chemical group quinone, while the number 10 refers to the isoprenyl repeats (CH_2_=C(CH_3_)−CH=CH−) in the tail of its chemical structure (Figure 1). The number of isoprenyl repeats varies (6–10) between species; for example, Coenzyme Q10 is the predominant form in humans, whereas Coenzyme Q9 is the predominant form in rats [2,3]. Such differences in the number of isoprenyl repeats affect the chemical properties of this molecule, such as mobility, autoxidizability, and the interaction with membrane proteins, and hence the stability within the mitochondrial membrane [4].

Coenzyme Q_10_ is a component of the electron-transport chain, which is responsible for the generation of adenosine triphosphate (ATP) molecules from aerobic cellular respiration [1]. In point of fact, 95% of ATP in the human body is generated by aerobic respiration [5]. Therefore, the concentration of coenzyme Q_10_ is very high in the functional organs with high ATP demand, such as the kidney, the heart, and the liver [6]. 

Coenzyme Q_10_ has been used as an attractive intervention approach in a wide-range of pathological diseases or disorders, such as cardiovascular disease [7,8], diabetes [9,10], kidney disease [11], Parkinson’s disease [12,13], Huntington’s disease [14], cancer [15,16], and infertility [17,18]. The efficacy of coenzyme Q_10_ in treating such diseases has allowed scientists in the field to study its safety via controlled-clinical trials. According to these studies, it has been shown that the maximum safe dose for coenzyme Q_10_ is 1.2 g per day; however, some short-termed clinical trials have used 3.0 g per day [19]. In addition, coenzyme Q_10_ at 2.4 g per day was found to be safe, as it was well-tolerated by patients with Huntington’s disease [20]. 

Testosterone is a sex steroid hormone derived from cholesterol [21]. In human males, about 95% of testosterone is synthesized in the testis, and the remainder (≈5%) is synthesized by other organs, mostly the adrenal gland [22]. The testis contains two types of cells: Sertoli and Leydig cells. Testosterone is primarily synthesized in Leydig cells, which are stimulated by the luteinizing hormone (also called lutropin) [23], which is a glycoprotein hormone secreted from the gonadotropic cells in the pituitary gland in response to the gonadotropin-releasing hormone [24]. 

Testosterone mainly acts in two ways: (1) by activating the androgen receptor itself, or after conversion to the active form (5-α-dihydrotestosterone) by 5-α-reductase; and (2) by conversion into estradiol (the most important estrogen in reproductive function health in females) by the aromatase enzyme [25,26]. Testosterone binds the androgen receptor mainly in the liver, muscles, and adipose tissues. In the liver, testosterone enhances protein synthesis, while in muscles, testosterone enhances muscle mass [27]. In the brain and bone, testosterone is converted by aromatization into estradiol, which binds to estrogen receptors [28]. Testosterone acts in the brain by stabilizing mode, enhancing libido, and improving cognitive function [29,30]. In skin, testosterone binds to the androgen receptor in its active form 5-α-dihydrotestosterone and enhances the growth of hair [22]. 

In addition, testosterone is one of the most important factors that has been found to modulate well-being and general health in males [24,31]. Lower levels of testosterone in human males has been found to be correlated with various diseases and disorders, such as diabetes [32], osteoporosis [33], bone loss [34], and infertility [35]. Therefore, there are many research studies that have revealed the influence of several bioactive supplements on testosterone levels in males. 

Enhancing testosterone production in males is still an imperative goal for many scientists in the field. This intention is attributable to the important role of testosterone as the main sex hormone in males [36]. Up to now, there have been more than 30 research studies that have linked coenzyme Q_10_ and testosterone. Such a link is attributable to the potent ubiquitous biological role of coenzyme Q_10_ as a vital member in the ATP generation process, as well as its powerful antioxidant activity, which is logically thought to have positive effects on testosterone production, and hence on male infertility in particular. However, this link has neither been narratively or systematically deliberated. Therefore, the present work systematically reviews and summarizes the impact of coenzyme Q_10_ supplementation on testosterone levels. To present this contribution, the Scopus, PubMed, and Web of Science databases were searched using the keywords “coenzyme Q_10_” versus “testosterone” for English language articles (full-texts or abstracts) from November 1978 through to October 2018. Relevant articles were also reviewed and included to present a complete systematic and comprehensive discussion.

## 2. Effect of Coenzyme Q_10_ Supplementation on Testosterone

The research studies to date that demonstrate a direct effect of coenzyme Q_10_ on testosterone are summarized in Table 1. These studies were conducted on both human and animal populations. Most of the time, the human studies recruited a group of male patients (e.g., infertile) where enhancing testosterone is part of disease management. Among this study lane, only one study was conducted on females. The last three studies in the table are reproductive toxicity studies, which measured the influence of coenzyme Q_10_ on testosterone levels after testicular injury by a chemical toxicant. 

The dose of coenzyme Q_10_ in human studies ranged from approximately 200 to 900 mg per day for about 2–12 months duration, while the utilized dose in animal studies ranged from ≈10 mg kg^−1^ day^−1^ to ≈500 mg kg^−1^ day^−1^ for approximately 5–96 days duration. As a supplement among these studies, coenzyme Q_10_ was obtained from different industrial and pharmaceutical companies from different countries (Japan, USA, Canada, Egypt). In accordance with this, whether the manufacturing quality of coenzyme Q_10_ affected the presented results in any given study is hard to predict. In terms of the form of treatment, coenzyme Q_10_ was given orally in all studies except for one study, in which it was given intraperitoneally. 

As a result, all studies conducted on human males generally presented an insignificant effect of coenzyme Q_10_ on testosterone. In addition, as opposed to the reproductive toxicity studies, the studies conducted on animals did not show any positive effect of coenzyme Q_10_ supplementation on testosterone. However, it is evident that coenzyme Q_10_ is able to counteract reproductive toxicity induced-testosterone depletion.

## 3. Mechanistic Studies

Specifically, luteinizing hormones regulate 17β-hydroxysteroid dehydrogenase expression, which converts androstenedione, a testosterone precursor, to testosterone [48]. The produced testosterone is transferred to Sertoli cells to induce spermatogenesis [49]. The effect of coenzyme Q_10_ on luteinizing hormones has been revealed in a number of human studies. A randomized placebo-controlled study showed that no significant changes in serum concentrations of the luteinizing hormone, dihydrotestosterone, or sex hormone binding globulin were observed between the intervention (oral coenzyme Q_10_ supplementation at 200 mg day^−1^ for 21 weeks) and the control group [37]. Alternatively, when supplemented at 300 mg day^−1^ for 26 weeks in infertile men, serum levels of luteinizing hormone deceased significantly in comparison with the control [38]. Moreover, an open-label prospective study has shown that infertile men with idiopathic oligoasthenoteratozoospermia treated with coenzyme Q_10_ at 900 mg day^−1^ for 3, 6, 9, and 12 months had lower levels of luteinizing hormones [39]. In fact, the meta-analysis conducted by Lafuente [50] revealed that supplementing infertile men with coenzyme Q_10_ does not increase the rate of pregnancy.

As explained above, given that the luteinizing hormone is the main stimulant of testosterone production, then the observed insignificant or negative influence of coenzyme Q_10_ supplementation on testosterone may be due to the insignificant or negative effects of coenzyme Q_10_ on the luteinizing hormone. However, more human studies seem valuable to confirm such blunted or negative effects. 

To date, almost all chemically induced reproductive toxicity studies that link coenzyme Q_10_ and testosterone have been conducted on male rats. In all of these studies, coenzyme Q_10_ was found to counteract the reduction in testosterone levels. Sodium arsenite, isoproterenol drugs, and aluminum chloride are examples of the chemical toxicants utilized to induce reproductive toxicity in rats, and thereafter to assess the effectiveness of coenzyme Q_10_ in ameliorating testosterone depletion.

In fact, the most evident chemical property of the used chemical toxicants that share the ability to induce reproductive toxicity, is that they are vigorous oxidizing inducers. These toxicants are able to induce the generation of reactive oxygen species, or oxygen radical species, such as the hydroxyl (OH^•^) and superoxide ion (O_2_^•−^) radicals, in cellular systems, and deplete the molecular and enzymatic antioxidant defense mechanisms [47], leading to random oxidative injury for cell components such as proteins, lipids, and nucleic acids [51,52]. For example, arsenic was found to form reactive oxygen species under physiological conditions via a direct-chemical binding with the thiol group (–SH), which for example, alters the cellular reservoir of glutathione, and in return exacerbates the level of oxidative stress and lipid peroxidation [53]. In addition, arsenic has been found to oxidize lipoic acid, an antioxidant coenzyme for dehydrogenase enzymes, by binding covalently and irreversibly to its free thiol groups [54,55]. Likewise, aluminum chloride was found to induce, at least in part, reproductive toxicity in male rats by decreasing the gene expression of antioxidant enzymes (catalase, superoxide dismutase, glutathione reductase) and reducing the content of glutathione, which consequently enhances the magnitude of oxidative injury. 

In general, oxidative stress and lipid peroxidation were found to diminish the function of the cell [21,56]. Therefore, counteracting this chemical oxidative mutilation should enhance cell function and cell recital [57]. Specifically, the accumulation of free radicals, and hence the oxidative injury in Leydig cells in the testis by a given chemical oxidant, may deteriorate their response and performance to synthesize testosterone. For example, it has been shown that aluminum chloride downregulated the gene expression of 3β-hydroxysteroid dehydrogenase, cholesterol side chain cleavage enzyme, and steroidogenic acute-regulatory protein [47]. In addition, arsenate treatment was found to reduce gene expression of the main enzymes (e.g., cytochrome P450 side-chain cleavage enzyme and 3β-hydroxysteroid dehydrogenase) in testosterone synthesis [58].

Indirectly, zinc has been identified as an important activator for the antioxidant enzyme superoxide dismutase [59,60]. Aluminum chloride was found to reduce zinc content in testis [47], which may aggregate the level of oxidative stress, and hence the level of oxidative injury, via reducing the efficacy of superoxide dismutase. Accordingly, it can be suggested that coenzyme Q_10_ supplementation is an effective intervention in such reproductive toxicity conditions.

Coenzyme Q_10_ has been identified as having potent antioxidant activity [61,62,63]. As evaluated by 2,2-diphenyl-1-picrylhydrazyl, coenzyme Q_10_ exerted strong free radical scavenging activity [64]. In addition, coenzyme Q_10_ was found to enhance the human nuclear factor (erythroid-derived 2)-like 2, which is a transcription factor that regulates the expression of certain antioxidant proteins [64,65]. Moreover, coenzyme Q_10_ attenuated H_2_O_2_-induced lipid peroxidation as evaluated by inhibition of malondialdehyde, an end product and a biomarker of lipid peroxidation [64,66]. Furthermore, coenzyme Q_10_ protected against cadmium-induced oxidative damage in Wistar albino rats [61]. I addition, ubiquinol (the reduced form of coenzyme Q_10_) supplementation prevented deprivation in coenzyme Q_10_, and decreases in paraoxonase enzyme activity [67] and reactive oxygen species content, during physical exercise [68]. Specifically, coenzyme Q_10_ supplementation was found to significantly suppress testicular oxidative stress and lipid peroxidation, and restore the antioxidant defense mechanism [45], which in turn can counteract chemical oxidative injury and maintain the function of Leydig cells to produce testosterone [69]. 

## 4. Conclusions and Future Perspectives

In conclusion, thus far the studies conducted on human males generally reveal an insignificant effect of coenzyme Q_10_ supplementation on testosterone levels. Similarly, the studies conducted on animals, rather than the reproductive toxicity studies, did not show positive effectiveness of coenzyme Q_10_ on testosterone. However, coenzyme Q_10_ supplementation was found to counteract testosterone reduction induced by chemical reproductive toxicants, mainly by counteracting the destructive effect of the generated pro-oxidants. In addition, according to the peer-reviewed literature in this specific context of research, studies performed on human males have revealed no beneficial effects of coenzyme Q_10_ supplementation on infertile men. Thus, dietary supplements containing much lower doses may not have any influence on the studied subjects.

This summary provides a specific intention for health care providers, particularly physicians, toward using coenzyme Q_10_ as a synergistic supplement with drug or chemical-induced reproductive toxicity. However, enhancing testosterone may require alternative therapeutic strategies, rather than coenzyme Q_10_ supplementation. Nevertheless, collectively these findings and suggestions require further confirmation. 

## Figures and Tables

**Table 1:** A summary of the main research studies conducted on coenzyme Q_10_ and its reported effects on testosterone.

Source	Dose (Mode of Treatment)	Duration	Study Population	Effect on Testosterone	Ref.
Coenzyme Q_10_ (Bio-Quinon Q10)	200 mg day^−1^ (orally)	21 weeks	Patients with hormonally untreated carcinoma of the prostate	(±)	[37]
Coenzyme Q_10_ (Kaneka, Osaka, Japan)	300 mg day^−1^ (orally)	26 weeks	Infertile men	(±)	[38]
Coenzyme Q_10_ (Nutri Q10, Nutri Century, Toronto, ON, Canada)	900 mg day^−1^ (orally)	12 months	Infertile men with idiopathic oligoasthenoteratozoospermia	(±)	[39]
Coenzyme Q_10_	Therapeutic dose (orally)	3 and 6 months	Patients with idiopathic oligoasthenospermia	(±)	[40]
Coenzyme Q_10_ (Nature Made Pharmaceutical Company, Mission Hills, CA, USA)	200 mg day^−1^ (orally)	8 weeks	Patients with polycystic ovary syndrome	(−)	[41]
Coenzyme Q_10_ (Nutralife Co., Richmond Hill, Canada)	125, 250, and 500 mg kg^−1^ day^−1^ (orally)	96 days	Bilateral orchidectomized male mice	(±)	[42]
Coenzyme Q_10_ (Nutralife Co.)	125 and 250 mg kg^−1^ day^−1^ (orally)	96 days	Gonadectomized male mice	(−)	[43]
Coenzyme Q_10_	10 and 20 mg kg^−1^ day^−1^ (orally)	2 months	Male ostriches	(±)	[44]
Coenzyme Q_10_ (Sigma Chemical Company, St. Louis, MO, USA)	10 mg kg^−1^ day^−1^ (intraperitoneally)	5 days	Male rats with sodium arsenite-induced reproductive toxicity	(+)	[45]
Coenzyme Q_10_	10 mg kg^−1^ day^−1^ (orally)	20 days	Male rats with isoproterenol-induced reproductive toxicity	(+)	[46]
Coenzyme Q_10_ (Arab Co. for Pharmaceuticals & Medicinal Plants, Cairo, Egypt)	10 mg kg^−1^ day^−1^ (orally)	10 weeks	Male rats with aluminum chloride-induced reproductive toxicity	(+)	[47]

(+) Increase; (−) Decrease; (±) No effect.
